# Privacy revisited? Old ideals, new realities, and their impact on biobank regimes

**DOI:** 10.1007/s10202-011-0094-x

**Published:** 2011-06-28

**Authors:** Arndt Bialobrzeski, Jens Ried, Peter Dabrock

**Affiliations:** Faculty of Philosophy and Department of Theology, Systematic Theology II (Ethics), PRIVATE Gen Project, Friedrich Alexander-University Erlangen-Nuremberg, Kochstraße 6, 91054 Erlangen, Germany

## Abstract

Biobanks, collecting human specimen, medical records, and lifestyle-related data, face the challenge of having contradictory missions: on the one hand serving the collective welfare through easy access for medical research, on the other hand adhering to restrictive privacy expectations of people in order to maintain their willingness to participate in such research. In this article, ethical frameworks stressing the societal value of low-privacy expectations in order to secure biomedical research are discussed. It will turn out that neither utilitarian nor communitarian or classical libertarian ethics frameworks will help to serve both goals. Instead, John Rawls’ differentiation of the “right” and the “good” is presented in order to illustrate the possibility of “serving two masters”: individual interests of privacy, and societal interests of scientific progress and intergenerational justice. In order to illustrate this counterbalancing concept with an example, the five-pillar concept of the German Ethics Council will be briefly discussed.

## Abstract (de)

Biobanken, die Körperproben, Krankenakten und lebensstilrelevante Angaben miteinander verknüpft aufbewahren, müssen zwei sich ausschließende Ziele verfolgen: Zum einen soll die öffentliche Wohlfahrt mittels möglichst schrankenlosem Zugang für die medizinische Forschung gesteigert werden, zum anderen müssen die restriktiven Einstellungen von Körperprobenspendern gegenüber der Privatsphäre respektiert werden, um deren Bereitschaft zur Studienteilnahme zu erhalten. Im folgenden Artikel werden neuere bioethische Entwürfe kritisch beleuchtet, die den sozialen Wert geringer Privatsphärenerwartungen betonen. Es stellt sich dabei heraus, dass weder utilitaristische, noch kommunitaristische oder klassisch libertäre Ethiken den beiden oben genannten Zielen gerecht werden können. Stattdessen wird aufgezeigt, dass John Rawls Differenzierung von dem „Guten“ und dem „Rechten“ genau dies leisten kann: sowohl dem gesellschaftlichen Interesse an wissenschaftlichem Fortschritt und Generationengerechtigkeit als auch dem individuellen Privatsphäreninteresse entsprechen zu können. Die Tragfähigkeit dieser Differenzierung wird abschließend an dem Beispiel des Fünf-Säulen-Konzepts des Deutschen Ethikrats illustriert.

## Agents of change

In the course of advancing genomic research, not merely critical opinions have been raised, which regard the potential of this scientific endeavor with skepticism, but rather statements, which are also directed in the opposite direction and express dissatisfaction with research restrictions, which offer a conditional understanding of autonomy and privacy as well. Accordingly, they demand to dissolve the current privacy regimes, which were derived from the Hippocratic Ethic and informed consent, and to adapt them to new development. For example, it is thus advised to relax the confidentiality obligation of a doctor, in order to enable researchers, who are not bound by any mutual trust toward the patient, to have direct access to physical specimens and sensible medical data (Lunshof et al. 2008a). Thereby it is often reasoned that not merely the private sphere of patients or specimens require protection, since they are essential for a self-determined life, but rather medical progress must also be protected to the same degree as a globally public good (Knoppers and Fecteau 2003). Without a significant increase in the participation of patients and their specimens in genomic-based research, developments in the discipline of biomedicine will not be satisfactorily achieved in the future (Harris 2005; Schaefer et al. 2009; Herrera 2003). Since the protection of the private sphere and impetus toward progress cannot be reconciled with each other by implication, but rather must be counterbalanced, among others, Ruth Chadwick proposed a further development of ethical standards that is appropriate to scientific advancement, which would lead to fewer restrictions. This is what she proposed:

As we hold the view that ethical thinking evolves alongside science, we argue that new models are needed to offer robust moral guidance while keeping the reality of a dynamic science in mind. (Lunshof et al. 2008b:406)

It seems obvious that complex clinical diseases could be better investigated and subsequently more effectively treated if personnel who are authorized to access data and physical specimens would be expanded. However, whether a relaxation of the Hippocratic Ethic must accompany this, so that confidentiality obligations and thereby respect for private spheres as basic prerequisites of an autonomous life would have to relinquished in the future, certainly requires further discussion.

The underlying basic conflict here between individual freedom and collective welfare permits different solutions. To begin with, two basic concepts will be presented in the following: One is utilitarian solutions, which broadly takes aim to achieve the greatest possible benefit for the largest possible number of people; the other is communitarian solutions, which concentrate on the benefit and values of individuals in the collective, from whom it is to be determined. In each case, at the conclusion of the delineation of these positions, inquiries regarding these conceptions will be named from a liberal viewpoint on one hand that insist upon the value of freedom as the basis of individual benefit; on the other hand that take into account questions of justice and thus keep an eye on the total societal benefit. Finally, a procedural approach will be presented, the so-called 5-pillar model of the German Ethics Council, which is intended to guarantee the named values and criteria in practice.

## Utilitarians and the duty of research participation

Since many years ago, arguments have been put forward from several utilitarian thinkers, who sought to enforce the participation in medical experiments as a moral or even legal obligation. The respective justifications vary as well as the respective proscribed degree of obligation, so that in the following discussion, the respective positions will be presented in the sequence of an increasing degree of obligation.

At first, the bioethicist John Harris is mentioned, who represents in a dual manner the obligation for participation in biomedical research (Harris 2005). At first he indicates that there exists a generally accepted moral obligation not to injure other living creatures. This culminates in the obligation to participation in research since an international abstinence would thereby injure people in that it enables diseases to persist, which could be prevented or minimized through a broadly based participation in medical research. On the other hand, the participation in medical investigations is a commandment of fairness in that everyone has benefited in one form or another from the results of earlier times. Accordingly, on the basis of reciprocity, everyone has the obligation to act for the benefit of future generations. Non-participation can be compared with unfair freeloading, since one realizes the benefits of previous research, but one abdicates responsibility for the benefit of others or for current research. In this connection, Paragraph 5 of the Helsinki Declaration in the version of 2000 caused some irritation which evaluated the benefit of the individual higher than the value of the collective.^1^ Thereby it was assumed that everyone knew best for himself what is conducive to his benefit. But Harris disputes exactly this point and he refers to the behavior of smoking, drug abuse, and selfless altruism, which are enjoyed against one’s own best interest. Thus, one should not place the collective interest above that of the individual only on the basis of a new balance, but rather also assume that participation in a study can also be of interest of the participants, even if they are not always convinced of it. Above all, there are many examples, such as the general stipulation to wear safety belts, pay taxes, and attend school, whereby collective and individual interests are combined through coercion and thus without consideration of discrepancies of individual objections (Harris 2005).

While the bioethicist John Harris derived a moral obligation to participate in studies from the human obligation for assistance and a necessary intergenerational justice, G. Owen Schaefer, Ezekiel J. Emanuel, and Alan Wertheimer argued from an economical viewpoint. The three researchers, who accompany the work of the largest, global research hospital in the USA from a bioethical perspective, advocate the definition of biomedical research as a public resource. They state that the non-participation in medical research is recognizable as ethical, while the participants undergo a moral verification, whether they are aware of the personal risks and burdens (Schaefer et al. 2009). Similar to John Harris, Schaefer et al. make the effort to reverse the burden of proof and court the moral value as much as possible that those who refuse to participate in research must justify themselves. Thereto, they consult a generally accepted definition of a public good in economics, which they regard to be generated by biomedical research: On one hand, it is something that does not lose its value through usage by other persons. On the other hand, it is something that is practically impossible to prevent its usage by other people. Conversely, a good is then private, if its value has been diminished through its usage by another person, or it is realistically possible to limit its usage to certain people and exclude others from using it. Thereby it is not relevant in their view, whether a public good has been produced privately or publicly, but rather who finally benefits from it. However, the problem is that there is practically no incentive to generate public goods normally, even if the individual usage is greater than the individual effort. Also nobody can be excluded from a public good, because the individual contribution thereto does not matter; this usually leads to the fact that the general public remains underprovided with public goods. Biomedical research stands exactly before this dilemma and it therefore demands more engagement of citizens. Thus, Schaefer et al. propose in fact no general legal obligation for participation in studies, but a moral prima facie obligation: It is expected from everyone to participate in studies, in order to assist the development of the public good of biomedical knowledge, which contributes to the public good of general health provision. Only those who can demonstrate convincing reasons can be excused from the moral obligation and refuse participation (Schaefer et al. 2009).

However, critics of this position indicate that in addition to more study participants, an increase in the participation of doctors is also needed, who generally are reticent to participate on the basis of their work load (Greene et al. 2009). Furthermore, the values that are generated are partially questionable, since not every study is clinically or societal culturally relevant (Katz 2009; Folayan et al. 2009). Also, a basic health provision that is not open to everyone in several countries may not require the participation of everyone on the basis of justice. Furthermore, employers should make their employees available for participation, in that otherwise sensitive wage reductions will occur for those with a low income; no one would accept this. Minorities, who have had bad experiences in the past with medical research and who continue to talk about them, must be informed about current protective measures, which are also bestowed upon them. Biomedical companies should attend more to the benefits for participants, for example, in the form of medical treatment, learning, and employment opportunities; then the recruitment would work better (Powell et al. 2009). Thus, on the basis of multiple obstacles, it can no longer be claimed that the deficient expansion of biomedical research is mainly caused by a fundamentally distinctive antipathy to participation.

A much higher degree of obligation is represented by the research ethicist C.D. Herrera. While the social philosopher Michael Walzer relies on the intuition of the average citizen for moral obligations, which would render public coercion obsolete for individual good deeds for the general public (Walzer 1983), by contrast, the research ethicist Herrera emphasizes the principle of universal obligation of everyone in a civil society, precisely that one cannot rely solely on an ethic of social obligation, but rather the principle must also be legally safeguarded. Even if an implementation of participation incentives should not be waived, ignorance of social obligations can be punished. Approved methods, such as the informed consent, could continue to be used, in order to maintain leeway within a system of obligation. Thereby one would not merely comply with justice, but also with the pursuit of autonomy (Herrera 2003).

In order to place the questions in perspective, whether the protection of individual autonomy or rather the development of collective benefits should be the guideline of the decision to participate, apart from all interesting, academic special debates, legal standards must be observed in pluralistic societies. With regard to this challenge, John Rawls introduced the central differentiation between “just” and “good” (Rawls 1988).

Each person possesses an inviolability founded on justice that even the welfare of society as a whole cannot override. For this reason, justice denies that the loss of freedom for some is made right by a greater good shared by others. It does not allow that the sacrifices imposed on a few are outweighed by the larger sum of advantages enjoyed by many. Therefore, in a just society, the liberties of equal citizenship are taken settled; the rights secured by justice are not subject to political bargaining or to the calculus of social interests. (Rawls 2005:3–4)

Thus, Rawls argues that societal culturally a common consensus can never be postulated over worthwhile goals and preferences of lifestyle. But since a peaceful coexistence in spite of different concepts and goals must be possible, at least an “overlapping consensus” (Rawls 1987) can be sought, which must be implemented, beginning with the framework of a basic law up to pragmatic, individual, statutory standards. Within this citizens then achieve certified rights. The “good” has indeed a high motivational power for the individual, but it may be linked to individual preferences and philosophies that are not shared by all citizens; therefore in doubt, it may have a disadvantage, with respect to their legal enforcement. What is decisive is thus that the “good” can only be enforced under the rule of law, provided it does not interfere with the current legal concepts.

A legal obligation for participation in medical research may only be implemented if it can achieve a verifiable and foreseeable very high “good” and prevents injury to the human population. Where this does not apply, it should be questioned critically, whether an obligation to participate exists in the sense of Schaefer et al., which serves a collective health provision, but burdens the individual, or corresponds to a concept of good (individual) life and the pursuit of important, collective interests, especially since it turns a legal-cultural standard on its head, since the burden of proof lies with the participant holdout and no longer with the person who is demanding the participation. Ultimately, medical research is not dispensed by the legal-theoretical principle, which permits the freedom of a person (may he be the researcher himself or the invalid who is hoping for a cure) to only go so far that he does not limit the freedom of another person, here in the image of the participant. In this respect, the reverse of the burden of proof that is propagated by Schaefer et al. must be rejected as long as it does not correspond to the concepts that were achieved in overlapping consensus.

## Communitarians versus Libertarians

Until now it is evident that utilitarian considerations orient themselves on the collective benefit as “good”, without thereby intrinsically assuming elementary standards of justice. This applies especially to the utilitarian reverse of the current burden of proof, which demands a justification obligation from those who require participation in the research, but who misalign this obligation to those who do not wish to make their data and specimens available to biomedical research. In this section, it is intended to demonstrate why communitarian requirements toward the limitation of the personal private sphere should also be observed critically.

Bartha M. Knoppers and Ruth Chadwick postulated that the technological advance in medical research to date has permitted successfully practiced ethical principles such as autonomy, privacy, justice, quality, and equity to now appear to be outmoded. This trend posed a challenge to bioethics to include other, up to now, ignored ethical criteria. Knoppers and Chadwick determined that a new form or a new understanding of reciprocity is being established in human genetic research, which assumes the requirements of researchers as well as the requirements of the donors of physical specimens and personal data. Thus, not only more recognition for participation in human genetic studies was achieved, but rather diverse, comprehensive participation agreements were presented, which not only could address augmented preferences for individual participation, but rather permit the researcher more possibilities to use the specimens for various purposes. Furthermore, it is emphasized that mutuality is also a value that must be considered, since families demonstrate a common genetic pool; upon discovery of life-threatening genetic factors, relatives should not be left in ignorance about shared genes that are co-responsible causes of illnesses. In addition, it must be considered that genetic information can be understood not as individual but rather as collective “good” of the family.

Against the background of this interpretation of the profound interwoven status of the individual in social and especially familial relationships, Knoppers and Chadwick ask the question whether a social obligation does not exist to demonstrate responsibility and to make his own data available to general medical research in solidarity with (potential) invalids. In addition, the question is asked of the general citizenry to consider the medical-ethical problem not merely from an individual viewpoint, but rather either from a perspective of an entire technical discipline, an interest group, or even simply from the viewpoint that is represented by the whole society and therefore would be related to positions that clearly exceed the individual limits of interest groups. Finally, both indicate the principle of universality that a general obligation is derived from the commonality of genetic material of all humans to treat the weakness of genetic material constructively, not least through participation in studies which serve all of mankind (Knoppers and Chadwick 2005).

The trend that was identified and supported by Knoppers and Chadwick away from rather individual-oriented ethical principles, such as autonomy, privacy, justice, quality, and equity to more collective principles, such as reciprocity, mutuality, solidarity, citizenry, and universality can be critically analyzed with respect to “freedom”. In order to achieve a differentiated view, two different understandings of freedom, presented by the political theoretician and social philosopher Isaiah Berlin, can be used to investigate the communitarian approach since the question of health and research, which serves it, are ultimately also questions of freedom.^2^ With his binary definition of *positive* and *negative freedom*, he created a multi-received (Carter et al. 2007; Miller 2006) categorical differentiation (Carter 2007), with which a complete series of freedom definitions can be differentiated and hence those that indicate to date a heuristic value in the question of emphasizing an individual right to privacy and the general, i.e., collective right to health provision. In connection with the philosopher Beate Rössler who differentiated informational, decisional, and local privacy (2005:9), a further step can be taken and the differentiation of positive and negative privacy can be extracted from the relationship of autonomy and Berlin’s definition of positive and negative freedom. Since freedom is twofold according to Berlin and from Rössler’s point of view, a fundamental condition of the realization of (twofold) freedom is autonomy; therefore, the existence of a yet to be defined private space is, in turn, de facto a prerequisite (Rössler 2005:9–10). In the following section, one can thus speak of negative as well as a positive private sphere. The relationship between freedom, autonomy, and privacy was explained by Rössler as follows:

“[W]e regard privacy as valuable because we regard autonomy as valuable, and because autonomy can only lived out in all its aspects and articulated in all its senses with the help of the conditions of privacy and by means of rights and claims to privacy. If the *telos* of freedom is conceived as being able to lead an autonomous life, then spelling out the conditions for such an autonomous life brings to light that civil liberties alone are not sufficient for the protection of autonomy, but that autonomy is reliant upon these civil liberties being substantialized in rights and claims regarding the protection of privacy. A person’s autonomy can be violated or impaired in ways that do not directly bear upon the civil liberties themselves, and it is because of this possibility that people are dependent in their autonomy upon the protection of privacy.” (Rössler 2005:9–10)

It is now imperative to further investigate this relationship between positive and negative freedom on the one hand and positive and negative privacy on the other hand for the sake of a critical examination with the self-designated communitarian position of Chadwick, Knoppers et al.

Concisely worded, Isaiah Berlin defined positive freedom as a *freedom to* something, while negative freedom was paraphrased by him as a *freedom from* something (Berlin 1990:131). The positive freedom thus signifies possibilities that arise in the sense of realizing freedom to do or become something and thereby to self-actualize. It is derived from the need of the individual to fashion his own life and to make decisions dependent on himself instead on external factors. Berlin formulated:

I wish to be the instrument of my own, not of other men’s, acts of will. I wish to be a subject, not an object; to be moved by reasons, by conscious purposes, which are my own, not by causes which affect me, as it were, from outside. I wish to be somebody, not nobody; a doer—deciding, not being decided for, self-directed and not acted upon by external nature or by other men as if I were a thing, or an animal, or a slave incapable of playing a human role, that is, of conceiving goals and policies of my own and realizing them. (Berlin 1990:131)

Applied to the context of biomedical research, positive freedom means to have the intrinsic motivation to make one’s own DNA and medically relevant data available to a biobank in order to minimize the morbidity and mortality of humans. This would be an instance of positive freedom, since this decision is taken without outside pressure and is the result of free, own decision. Additionally, the personal, private portion is not hidden, but rather implemented for the benefit of many in public, similar to utilization of political, positive freedom, introducing one’s self into public discourse, and the possibility of being able to utilize democratization or self-government.^3^ However, it appears problematic that a volunteer would deposit parts or substances of his own body in biobanks, if he could not determine their application, in any case not in their entirety and at all times. This would be equivalent to a limitation of positive freedom, because the right of self-determination finds its limit in the content of the signed informed consent and thus the complete self-determination is no longer given as a fundamental condition of positive freedom. In fact this is less the case with a detailed informed consent, where an explicit consent must be given to one concrete research and researchers must be bound to the concrete wishes of the volunteer, but the alternative form of broad consent and open consent or generic consent offer the researchers a broader latitude in which they no longer must coordinate in detail with the volunteers and thereby can significantly limit positive freedom.

Admittedly, Berlin had determined that the idea of positive freedom can also be abused. In fact, different instances come into consideration that can direct one’s own life, such as the “nature” of mankind, one’s own understanding, a leading idea, and the “elevated” self. However, logical reasoning is usually given preference above all else that is defined as being irrational. Mankind is then broken down into a rational, autonomous and a (to some extent) irrational, “empiric” heteronymous self; both parts can be pitted against each other. The higher autonomous self must defend himself against the lower, heterogeneous nature; the latter must be disciplined and domesticated (Berlin 1990:132).

A problematic aspect of this splitting by Isaiah Berlin is consequently the possibility of being able to legitimize obligations on the rational level with emphasis on rationality which can no longer clearly be differentiated from coercion. Berlin phrased in the following way:

This renders it easy for me to conceive of myself as coercing others for their own sake, in their, not my, interest. I am then claiming that I know what they truly need better than they know it themselves. (…) Once I take this view, I am in a position to ignore the actual wishes of men or societies, to bully, oppress, torture them in the name, and on behalf, of their ‘real’ selves, in the secure knowledge that whatever is the true goal of man (happiness, fulfillment of duty, wisdom, a just society, self-fulfillment) must be identical with his freedom—the free choice of his “true”, albeit submerged and inarticulate, self. (Berlin 1990:133)

In a clinical scenario, this could signify that if a moment of decision-making for or against participation in a study would cause a conflict between reason and moral intuition, the doctor as well as the researcher could persist in the primacy of rationality, be it derived from trust in communitarian values or be it derived from self-serving motives. If the moral intuition urged a rejection, since anxiety exists in the background about the misuse of donated DNA or data and the desire for negative freedom would be manifested, the doctor or researcher could propagate communitarian ideals on the level of understanding, such as solidarity and universality; he could thus promote a paradigm of positive freedom. Since in our cultural area, reason tends to be given the advantage and it awakens the appearance of self-evidence, it would be difficult to avoid well-intentioned arguments; thus, a dependent moment of decision would be created. This would be legitimized given that an object of suppression, if he were only more rational and enlightened, would exhibit a different behavior without coercion. “True motives” would be projected into the person to be coerced who is allegedly merely overshadowed by an empirical being; therefore they must be liberated through compulsory measures. An arbitrary situation would indeed not be created, because the communitarian, ethical framework should apply to everyone; however, the freedom of mankind would be curtailed, as described in a special manner by John Harris and C.D. Herrera.

The oppressive situation to be feared turns out to be relevant even by inspecting the argument of Bartha Knoppers and Ruth Chadwick. Thus, real dangers in fact exist that stigmatization, discrimination, and not yet foreseeable future dangers could appear for a volunteer, which are given through ever-improved possibilities for the interpretation of DNA, medical, and lifestyle-related data (Murray 1997). However, they could rapidly appear as improbable individual risks by certain interpretations of a doctor or researcher, which still appear to be acceptable with regard to higher values such as reciprocity, mutuality, solidarity, citizenry, and universality (Knoppers and Chadwick 2005). Those arguments permit a “reasonable” person no other choice but the participation in human genome research. Thus, it is not surprising that even Ruth Chadwick quite early posed the question, whether at least a moral obligation to participate in human genome studies should exist, and thereby proposed the same requirements as utilitarian bioethicists:

We also contend that the benefits of research could be shared more widely by those who profit, and that there is a duty to participate in research that could move medicine forwards on the basis of solidarity. It is questionable whether individuals should be free, from an ethical point of view, to refuse to help in an effort to relieve suffering for what could be regarded as trivial reasons, such as refusing to allow samples to be reused for research on drug abuse because of the disapproval of drug users. (…) [N]ow might be the time for a fresh ethical perspective. (Chadwick and Berg 2001:321)

Ruth Chadwick herself indicates significantly in a later article that was published together with Lunshof et al. (2008b) that the private sphere is often endangered by participating in human genome studies, but they seek simultaneously to reduce the need to protect the private sphere, in order to eliminate the greatest possible obstacles from scientific research.

If the private sphere is endangered, negative freedom is thus affected, which marks an especially protected zone; according to Beate Rössler, it represents a core element of liberal democracies (2005:9–10). The *negative freedom* in the sense of freedom from something signifies accordingly the absence of limitations, coercion, and hindrances by other people, especially by government authority (Berlin 1990:122–131):

By being free in this sense I mean not being interfered with by others. The wider the area of non-interference the wider my freedom. (Berlin 1990:123)

Even when people strive for other values in addition to freedom, such as justice, happiness, culture, security, and equality and would even accept relinquishing a portion of their freedom (and if one proceeds further than Berlin and must question more intently the material, ideal, and social conditions to enable this negative freedom), a lower limit of personal freedom must remain protected nevertheless by all means. Without it, people would be robbed of a nucleus, which empowers the development of those capabilities that they need for the achievement of their own goal. Therefore, a clear limit is absolutely necessary between the area of private life and that of public authority (Berlin 1990:123–124). With a reference to John Stuart Mill, he reminds us that there can be no progress without a private place of leisure:

[U]nless men are left to live as they wish ‘in the path which merely concerns themselves’, civilization cannot advance; the truth will not, for lack of a free market in ideas, come to light; there will be no scope for spontaneity, originality, genius, for mental energy, for moral courage. Society will be crushed by the weight of ‘collective mediocrity’. Whatever is rich and diversified will be crushed by the weight of custom, by men’s constant tendency to conformity, which breeds only ‘withered capacities’, ‘pinched and hidebound’, ‘cramped and warped’ human beings. (…) ‘All the errors which a man is likely to commit against advice and warning are far outweighed by the evil of allowing others to constrain him to what they deem is good.’ (Berlin 1990:127)

Transposed onto the handling of the private sphere in biobanks, negative freedom means that the DNA, which is stored in combination with phenotypic relevant data, can (but not must) lead to stigmatization and discrimination, on the basis of an unauthorized or undesired evaluation (Lemke and Lohkamp 2005), and therefore, a protection requirement normally exists for DNA donors. This protection requirement can also be designated as a negative private sphere and thus creates a heuristic tool, in order to be able to differentiate between voluntary participation and the need for protection; however, this leads to a dichotomy which should be resolved in the following section.

## Ethical gradualism versus dichotomies

In the consideration of individual and collective interests, after a critical review of approaches from communitarians, pragmatic utilitarians, and liberals, it is recommended to avoid dichotomy determinations and one-sidedness and thereby to revert to an ethical individualism, which is appropriate to the complexity of the material and which accommodates the multiple interests in equal measure. Before the background of Rawls’ theory of justice, a solution is offered to strengthen the positive freedom in an ethical manner and thereby to promote the “good”, but nevertheless to set a limit in the sense of negative freedom, as long as communitarian ideals do not include the legal overlapping consensus in a pluralistic society. Thus, such a restraint would fulfill Paul Ricoeur’s dictum that the goal of human ethical practice is “aiming at the ‘good life’ with and for others in just institutions.” (Ricoeur 1992:172)

As an instrument of ethical gradual differentiation of privacy regimes, Niklas Luhmann`s investigation of temporal, factual, and social dimensions of human communication is suitable (Luhmann 1997). Every potential candidate of a clinical study would accordingly stand in a relationship to the researcher, the biobank as well as to society, of which he is a part. The factual dimension would be to question, e.g., to which research purpose the participation will lead, besides the temporal dimension, which amount of time the study will claim, in addition, the social dimension, which asks about the social consequences, such as the personal gain for one’s own health, but also retains the theoretical possible negative social outcome for employment and family. Under the aspect of the factual dimension, it can be asked whether a clinical study, which invites participation, fulfills the criteria of effectiveness and efficiency as well as the benefit of the entire societal culture in the form of cost reduction as well as improvement in diagnosis, and therapy appears to be quite feasible (Wilson and Jungner 1968; Dabrock 2008). Especially significant for a gradual ethical consideration would be the social dimension, since in the case of genome research, it requires special, if not exclusive, risks versus other bio- or social markers, which could become concrete in stigmatization and discrimination with regard to partner selection, family planning, career decisions, and insurance coverage; hence, they require a specially intensive consideration (McNally et al. 2004). Such risks that are dependent on the design of the study but also from the scope of the authority to access data must then be carefully weighed with regard to human dignity which grants every person protection and respect, independent from his given or not given characteristics simply qua personhood (Dabrock et al. 2004), but also from the background of several European laws and guidelines like the EU Data Protection Directive (European Parliament, Council of the EU 1995), the European Anti-Discrimination Directive (Council of Europe 2000), the European Convention on Human Rights and Biomedicine (Council of Europe 1997), the European Convention for the Protection of Human Rights and Fundamental Freedoms (Council of Europe 2010), and diverse international biotechnology regulation recommendations (e.g. OECD 2009). Accordingly, if the potential of stigmatization and discrimination is minimal, e.g., the risk is minimal that one’s own autonomy will become limited, then lower standards are necessary and participation in the study would be morally desirable. However, such a moral desirability is not identical with a legal obligation. The former achieves the legitimization to support the participation in the study.

In order to motivate people to make their DNA available in the sense of positive privacy for the good of the entire society, incentives should therefore be set on the social level in different functional systems, for example, through an active turbo-charging of ethical preferred practices with positive connotations. Thus, for example, the DNA donations could be anchored in the social consciousness similar to blood donations as praise-worthy and thus attractive in the realm of civil society engagement. Furthermore, it would be worthwhile to ponder the act of participation not as research promotion, but rather to define it as one’s own research performance, in order to extend the social reputation of the scientists to the donors and thus to increase the attractiveness of participation. Thereby the juristic instrument would remain idle, in certain cases even unnecessary, since fluent changeovers from good to rights are happening sometimes. Only it must not be forgotten to continue to propagate the ideal of the good, since the society is ultimately dependent on it, in order to thus conceptually fortify its understanding of right with the good and to rely on their motivational power (Dabrock 2006). An understanding can be established therewith, which seeks viable visions, which integrate solidarity, reciprocity, and additional principles of personal responsibility for others, without hence objecting to a risk, to endanger individual freedom and to achieve a type of health obligation for everyone. Hence, it is permissible to promote a positive freedom on the fundament of considering negative freedom, which does not regard individual and collective interests as opposites, but rather as complimentary fertilizations. Therefore, in order to court personal engagement in research, it should be promoted, instead of creating moral or even legal pressure. Good reasons, which can create the appropriate discernment, do in fact exist. They do not inevitably take effect, but behind this openness, the possibility and mission of human decisions are demonstrated, in other words: human freedom.

## Anticipating a just and good biobank regime

It has become clear that utilitarian, communitarian as well as liberal approaches lead to one-sided measures and before the background of political liberalism in the person of John Rawls, an ethical gradualism can offer a way out of simplistic and dichotomic perspectives. This way out should serve the goal of supporting genome research on the whole to the benefit of individual patients as well as the public provision of health services, but simultaneously strengthen the protection of donors and participating patients, so that the interests of the collective as well as those of the individuals will be satisfied. But how must such gradualist perspectives be implemented and applied in a reasonable governance-perspective? A balance between the understanding of positive and negative freedom must thereby be established with the help of institutions so that ultimately the demands of a relativeness of the Hippocratic Ethic that was mentioned as the onset will be obsolete, and even result in the opposite, and nevertheless the medical and public health perspectives of genome research can be realized.

The opinion of the German Ethics Council for revision of biobank governance made institutional, theoretical, and practical suggestions, in order to balance the tension that has been mentioned. The intrinsic point of the impressive concept is that it is not composed of a single principle, but rather by dint of the alternating support of five dimensions—the text talks about five pillars—and suggests a procedural approach that enables latitude for medical research as well as respects individual, legally warranted protective standards. Thus, the five-pillar concept that was conceived by the German Ethics Council (2010) begins with the first pillar of an extension of the rule of confidentiality from doctors to every researcher and employee of a biobank, who has access to physical specimens and data, including a privilege to refuse to give evidence to the police as well as a confiscation prohibition. Hence, the so-called biobank confidentiality protects the personality rights and the informational self-determination, especially versus insurance companies, employers, and public authorities.

With the second pillar, the consent requirement is in fact retained, but simultaneously the possibility is guaranteed to be able to select between different degrees of agreement for research purposes. The samples and data will accordingly be determined for an explicitly defined research purpose, but they can also by mutual agreement be released from research limitations. In this instance, the limitation would be nullified for a single research project, a particular research facility or the time limit of the license term. Through the biobank confidentiality, it will simultaneously be ensured that the risk of misuse of specimens and data will be minimized and thus the control requirement of the participants will be reduced. The right of withdrawal can in fact be used, in order to remove one’s own specimens and data from the research, but simultaneously to refrain from an obligation to also destroy the contiguous research results. The possibility should be offered, with the consent of the donor, to be able to continue to use the research results, provided that one’s own data may be used only aggregated and without person-related data. Thus, the withdrawal volition of the donor as well as the concern of the researcher can be met at the same time to continue to use the data that have been gained to date and to render the scientific benefit not wholly obsolete. The outcome thereby is that the maturity of the donor himself to be able to determine the degree of risk to participate is set against paternalism.

The third pillar contains an obligatory involvement of ethics commissions for specimen and data collection without temporal and thematic limitations as well as the obligation for periodic evaluation of proprietary activities. When this recommendation has been implemented, it is guaranteed that ethically questionable research projects can no longer be performed. Should research be performed with person-related, non-anonymous specimens and data, which represent a massive invasion of personhood rights, then the ethics commission must grant in each instance a vote of approval prior to the performance, in order to maintain a high protective standard of personhood rights. The potential for misuse of specimens and data will thereby be appreciably reduced and thus the negative freedom of the donor will be strengthened.

The fourth pillar requires a persistent responsibility of the biobank for the security of specimens and data as of their admittance situation until their liquidation and destruction, since the biobank transgresses the bidirectional relationship between patient and doctor and must conduct an independent establishment of mutual trust on an institutional level.

The fifth pillar proceeds in the same direction: it requires a distinctive transparency especially for collections without temporal and thematic limitation, which make the storage and processing of specimens and data comprehensible at any time and thereby can meet the control interest of donors. If the available information is as meager as expected at the onset of such a collection, the transparency can satisfy the ongoing need to know about the biobank’s practice.

In summation, the model of the German Ethics Council first and foremost, in fact, cannot and will not set any moral impulses, which propagate a certain vision of good (or the promotion of genome-based research for the purpose of improving public health); instead, its strength however is the concentration upon the protective rights of the citizens, by spelling out the legal and organizational overlapping consensus and by strengthening trust in the participating institutions (Dabrock et al. 2011). By means of this procedural manner, research freedom will be strengthened in reality so that individual and public health can also benefit from it. Moreover, it enables an increased degree of protective rights and control possibilities, which reduce donor inhibitions and thereby convince more people to donate specimens and to ease previous restrictions for their usage or to dispense with them completely. Simultaneously not only the interests of the collective will be kept in focus, but rather the protective requirement of each individual will be strengthened as well as an elevate transparency, an improved data security and a more frequent involvement of the ethics commissions. Moreover, there remains, of course, much scope for a plural society, to develop effective concepts of good, which include the participation in genome research without too much pressure of moral obligations.

## Footnotes

1 In the latest version of the Declaration of Helsinki (World Medical Association 2008) the respective paragraph has changed only marginally. The individual welfare is not explicitly prioritised anymore over the interests of science or society. However, it is still generally mentioned that the wellbeing of the research subject stands above all other interests.

2 As an example, it could be asked: Shall a patient be liberated from a disease, and is this person dependent on other people who make use of their freedom to help?

3 Even though the definition of *negative* privacy as a protected sphere against interferences and intrusions of others aligns with Lars Ø. Ursin’s definition, our definition of *positive* privacy differs from his one because the sharing of medical information under the condition of confidentiality is not meant. Instead, we argue that *positive* privacy describes the fact that somebody intentionally discloses valuable private data for the benefit of others even though this information loses the private status in the end. Ursin defines a “distinction between *negative privacy* as the right to be left alone versus *positive privacy* (confidentiality) as the right to share information with someone without them passing it on.” (Ursin 2008:270).
